# Oestrogen-induced angiogenesis and implantation contribute to the development of parasitic myomas after laparoscopic morcellation

**DOI:** 10.1186/s12958-016-0200-y

**Published:** 2016-10-06

**Authors:** Ben-Shian Huang, Muh-Hwa Yang, Peng-Hui Wang, Hsin-Yang Li, Teh-Ying Chou, Yi-Jen Chen

**Affiliations:** 1 Department of Obstetrics and Gynaecology, Taipei Veterans General Hospital, No.201, Sec. 2, Shih-Pai Road, Taipei, 112 Taiwan; 2 Department of Obstetrics and Gynaecology, National Yang-Ming University Hospital, No.169, Siaoshe Road, Yilan, 260 Taiwan; 3 Department of Obstetrics and Gynaecology, School of Medicine, National Yang-Ming University, No.155, Sec.2, Li-Nong Street, Taipei, 112 Taiwan; 4 Institute of Clinical Medicine, National Yang-Ming University, No.155, Sec.2, Li-Nong Street, Taipei, 112 Taiwan

**Keywords:** Myoma, Parasitic myoma, Oestrogen, Aromatase inhibitor (AI), Selective progesterone receptor modulator (SPRM)

## Abstract

**Background:**

Iatrogenic parasitic myomas (PMs), caused by intra-corporeal power morcellation during laparoscopy is gradually increasing. However, the pathogenesis and medical treatment of PMs remain largely unelucidated.

**Methods:**

Laparoscopically-induced PM xenografted mouse model was conducted by xenografting human uterine myoma fragments into the abdominal cavity of SCID mice and hormonal manipulation was performed using this mouse model to demonstrate the role of oestrogen in the development of implanted PMs. Immunohistochemistry of oestrogen receptor α (ERα), progesterone receptor (PR), vimentin, vascular endothelial growth factor (VEGF), microvessel density (MVD) and Ki-67 index was performed and compared.

**Results:**

In the patient with PMs, ERα, PR, angiogenesis and proliferative property expression were upregulated in PM lesions compared to uterine myomas. In the laparoscopically-induced PM mouse model, implanted myomas had more steroid receptor expressions, angiogenesis and proliferative property compared with pre-xenografted or non-implanted myoma. Depletion of oestrogen in the ovariectomized (OVX) mice decreased laparoscopically-induced PM implantations. In comparison, the implantations of PMs were increased with additional E2 supplement. Hormonal manipulation in the PM mouse model, including AI, GnRHa and SERM groups, were compared and AI significantly decreased the implantations, steroid receptor, angiogenesis, cell density, and proliferative index of PMs compared with control group. Furthermore, GnRHa significantly decreased VEGF and MVD expressions compared with control group.

**Conclusions:**

These data highlight the crucial role of oestrogen in the development of laparoscopically-induced PMs and suggest that hormone manipulation may be a potential therapeutic agent.

**Trial registration:**

This protocol was approved by the Human and Animal Institutional Review Board of Taipei Veterans General Hospital (VGHIRB No 2014-10-002C on Nov. 17^th^, 2014; IACUC 2014-119 on Aug. 22^nd^, 2014).

**Electronic supplementary material:**

The online version of this article (doi:10.1186/s12958-016-0200-y) contains supplementary material, which is available to authorized users.

## Background

Uterine myomas (UMs) are the most common gynaecologic tumours, occurring in 40–70 % of women over 30 years of age [1]. For years, the only curative treatment for myomas is considered to be surgery including hysterectomy and myomectomy [2]. Laparoscopic procedures can be performed with decreased morbidity compared with exploratory laparotomy [3]. Steiner et al. introduced the first ‘electrical cutting device’ for laparoscopic removal of tissue from the abdominal cavity in 1993 [4]. However, power morcellation is certainly associated with tissue spreading per se resulting in intra-corporeal ectopic implants [5, 6]. The first case of a parasitic myoma (PM) after use of the laparoscopic morcellation was reported in 1997 by Ostrzenski [7]. With increasing frequency, laparoscopic and robotic-assisted myomectomies/hysterectomies are being performed in managing women with symptomatic myomas [5], a number of case reports were published that reported growth of PMs after morcellation.

The reported incidence of PMs after laparoscopic myomectomy was 0.20–1.25 % [8, 9]. Patients with PMs presented with multiple lesions and varying sizes (range, 0.8–30 cm). PMs largely occurred in the dependent part of the abdominal cavity, including intestines, peritoneum, omentum and port sites, and received abundant blood supply [8, 10–17]. Most women (78.3 %) presented with symptoms, such as abdominal or pelvic pain, dyspareunia, abdominal distension, abdominal pressure, urinary frequency and constipation [13, 14, 17–23]. In 10.1 % patients, debulking procedure, such as omentectomy, appendectomy, or bowel resection, was necessary to eliminate all PMs [18, 24, 25]. Thus, it is important to elucidate the pathophysiology of laparoscopic morcellator-induced PMs.

UM growth is dependent on the sex steroid hormones, oestrogen and progesterone [26]. Data from in vitro and animal models over decades suggest that oestradiol (E2) plays a central role in myoma growth via its receptor, oestrogen receptor α (ERα) [27, 28]. Most medical treatments reduce menstrual bleeding in patients with myoma, including gonadotrophin-releasing hormone agonist (GnRHa), aromatase inhibitors (AI), selective oestrogen receptor modulators (SERMs), progestins and selective progesterone receptor modulators (SPRMs) [1, 29, 30]. However, only GnRHa, AI and SPRMs can reduce both myoma volume and menstrual bleeding. Thus, it is hypothesised that exposure to oestrogen and progesterone could be a risk factor for the development of PMs [8, 31]. Until now, these evidences in managing PMs were based on case reports and small case series, which most received surgical treatment. Thus, the role of medical treatments for laparoscopically-induced PM remains largely unelucidated.

Due to the implantation and growth behaviour of PMs, we reasoned that oestrogen and angiogenesis might be involved in the development of PMs. In the present study, we simulated the laparoscopically-induced PMs xenografted mouse model to demonstrate the role of oestrogen-induced angiogenesis in the development of laparoscopically-induced PMs. Furthermore, we investigate the therapeutic effects of hormonal manipulation for PMs.

## Methods

### Study design

UMs of the proliferative phase were collected during myomectomy or hysterectomy from 7 premenopausal women with UMs without a history of using oral contraceptive or other hormonal treatments within 3 months, and one patient presented with PM after laparoscopic myomectomy (patients characteristics in Additional file 1: Table S1). This protocol was approved by the Human and Animal Institutional Review Board of Taipei Veterans General Hospital (VGHIRB No 2014-10-002C; IACUC 2014-119).

### Laparoscopically-induced PMs mouse model: xenografting human UM fragments into abdominal cavity of SCID mice

Firstly, five SCID mice without bilateral ovariectomy (OVX) were xenografted with UM fragments obtained from two patients (Case 2 and 3) (Additional file 1: Table S1). Because uterine myoma is also known as an oestrogen-dependent disorder, fresh UM samples were fragmented into 1–2 mm diameter sections under sterile conditions. The fragments were cultured in Dulbecco’s Modified Eagle’s Medium (DMEM) + Ham’s F12 (1 : 1) +10 % fetal bovine serum (FBS) supplemented with E2 (10^−9^ M) (Sigma-Aldrich, St. Louis, MO) for 4 h prior to xenograft into 8-week-old NOD-SCID female mice [32, 33]. One 1-cm longitudinal incision at the lower abdomen of SCID mice was made and ten UM fragments were implanted in the four quadrants of the peritoneal cavity. Before the wound was totally closed, the pneumoperitoneum needle (Surgineedle™, Covidien, US) was inserted into the abdominal cavity. The CO2 insufflation pressure was 4 mmHg, and the duration of insufflation was 10 min [34]. These mice were left untreated and sacrificed in 3 weeks after the xenograft. Both implanted and non-implanted fragments were harvested (xenograft and insufflation procedures in Additional file 2: Figure S1A & Additional file 3: Part 1).

### E2 treatment and OVX in laparoscopically-induced PMs model

Thirty SCID mice were equally grouped into OVX, control and E2 groups and were xenografted with UM fragments obtained from patient case 4 and 5 (Additional file 1: Table S1). Mice in OVX group received bilateral OVX two weeks prior the xenograft procedure (Additional file 2: Figure S1B). The others in control and E2 groups received the xenograft procedure without bilateral OVX. Mice in OVX (*n* = 10) and control (*n* = 10) groups were treated subcutaneously (S.C.) (100 μl, 0.9 % saline per week); mice in E2 group (*n* = 10) were treated with E2 S.C. per week (2.5 μg/ml estradiol in 100 μl 0.9 % saline, 10 μg/kg body weight; Sigma-Aldrich, US) [35]. The serum E2 levels were assayed at week 0 (just before the xenograft procedure and E2 treatment) and week 3 (just before sacrifice) (Additional file 3: Part 2). Laparotomic examination was performed and these mice were sacrificed 3 weeks after the xenograft procedures.

### Hormonal manipulation of laparoscopically-induced PM mouse model

Forty-eight mice xenografted with mixed UM fragments from patient case 6 and 7 (Additional file 1: Table S1) were equally grouped in to four groups according to the treatment protocols: control with 100 μl 0.9 % saline S.C. per week (*n* = 12) [35], AI (Letrozole; Femara® were prepared in 0.3 % hydroxypropylcellulose; mice were injected S.C. five times per week with letrozole 10 μg per mouse per day; Novartis, Switzerland) (*n* = 12) [36], GnRHa (Leuprorelin acetate S.C. 10 mg/kg per week; LEUPLIN®DEPOT 3.75 mg S.C. Injection, Takeda, Japan) (*n* = 12) [37], and SERM (Raloxifene hydrochloride, 0.1 μg S.C. per day; Sigma-Aldrich, US) [32].

Laparotomic examination was performed and these mice were sacrificed 3 weeks after the xenograft procedures.

### Histological and immunohistochemistry (IHC) analysis

The hematoxylin and eosin (H&E) and IHC staining, including ERα, PR, smooth muscle actin (SMA), Ki-67, vimentin, VEGF, and CD34, were performed and antibody characteristics are listed in the Additional file 1: Table S2. The H&E and IHC slides were independently examined by two observers. The cell density was determined on H&E sections and the immunohistochemical scores (IHS) was utilised based on the German ImmunoReactive score (Additional file 3: Part 3).

### Statistical analysis

Pearson *χ*2 or Fisher’s exact tests were used for comparison of dichotomous variables. The independent Student’s t -test or analysis of variance was used to compare the continuous variables between groups. All other data was analysed using the software SPSS (version 21; IBM Inc.)

## Results

### Case report: PMs possessed more ERα, PR and angiogenesis expressions compared with in situ UM

**Fig. 1:**
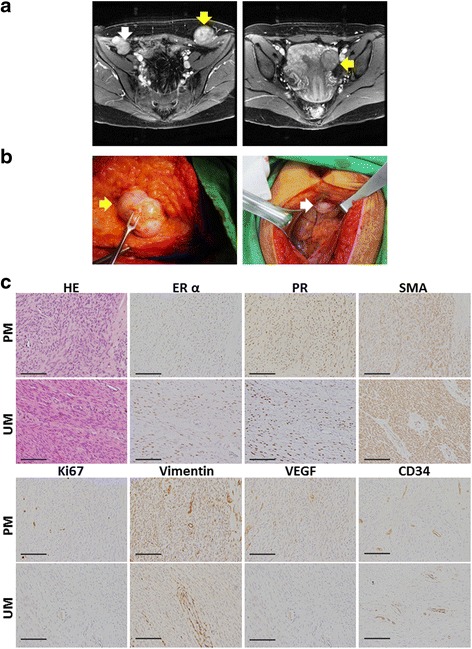
PMs presented with more cellularity, sex steroid receptors, proliferative property and angiogenesis expressions compared with UMs. **a** The imaging study of magnetic resonance imaging (MRI). (*Left*) one PM (*white arrow*) located at the level between peritoneum and fascia transversalis at right lower abdominal wall and another PM (*yellow arrow*) located at left lower abdominal wall on post-Gadolinium T1 weighted image. (*Right*) one UM (*yellow arrow*) located at left uterine wall on post-Gadolinium image. **b** Resection of PMs. (*Left*) One PM, 5*4 cm, (*yellow arrow*) located at previous trocar site; (*Right*) one 2*2 cm PM (*white arrow*) at sub-umbilicus for laparoscopy. **c** Staining for H&E, and IHC for ERα, PR, SMA, Ki67, vimentin, VEGF and CD34. Original magnification: ×400; the scale bars represent 400 μm. UM = uterine myoma, PM = parasitic myoma, ERα = oestrogen receptor α, PR = progesterone receptor, SMA = smooth muscle actin, VEGF = vascular epithelial growth factor

One 44-year-old female, G2P2, having a history of laparoscopic myomectomy with power morcellator 5 years ago, presented with chronic pelvic pain and one palpable left lower abdominal mass. The MRI image demonstrated with three soft tissue masses at previous trocar sites of the abdominal wall and laparoscopically-induced PMs was impressed (Fig. 1a). Laparotomic total hysterectomy and resection of abdominal wall lesions were performed (Fig. 1b). The pathological report of PMs disclosed benign UMs without atypia. Compared with in situ UM, PMs possessed more cellularity and Ki-67 index and more ERα, PR, vimentin, VEGF and CD34 expressions (IHS presented in Fig. 1c & Additional file 4: Figure S2).

### Sex steroid receptors and angiogenesis markers are associated with implantation of laparoscopically-induced PMs

**Fig. 2:**
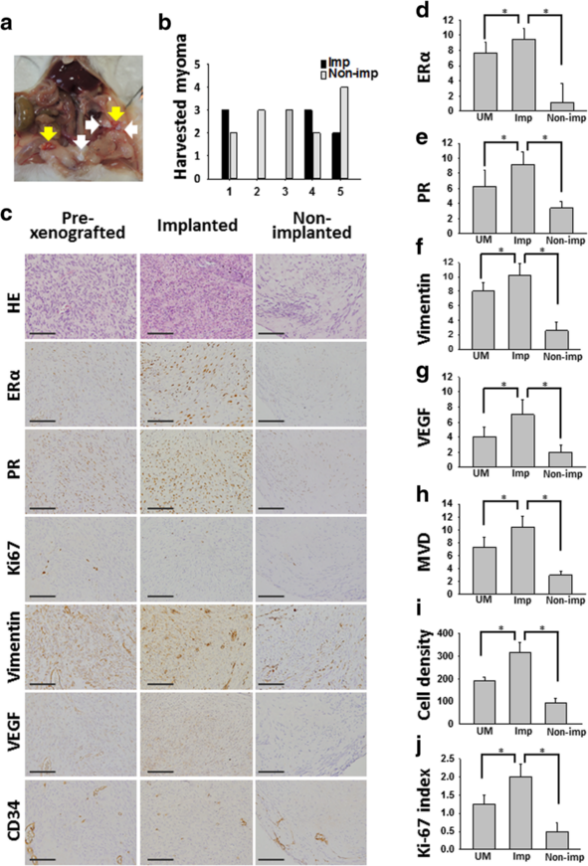
Implanted myomas presented with higher ERα, PR, vimentin, VEGF and MVD compared with pre-xenografted myoma and non-implanted myoma. **a** Implanted and non-implanted myoma fragments at sacrifice 3 weeks later. *Yellow arrows* indicate implanted myoma; *white arrows* indicate non-implanted myoma. **b** Implantations and non-implantations in each mouse at sacrifice. 3 of 5 SCID mice developed implanted uterine myoma. **c** H&E, and IHC stainings for ERα, PR, Ki67, vimentin, VEGF, and CD34. Original magnification: ×400; the scale bars represent 400 μm. **d**–**j** IHS of three groups. Implanted myoma presented with higher ERα, PR, VEGF, MVD cell density and Ki-67 expressions compared with pre-xenografted uterine myoma and non-implanted myoma. UM = pre-xenografted uterine myoma, MVD = microvessel density, Imp = implanted, Non-imp = non-implanted, *P < 0.05

We first investigated whether overexpression of sex steroid receptors (ERα and PR), angiogenesis and proliferative property occurred in PMs’ lesions. In the laparoscopically-induced PM mouse model, the mean implanted lesions were 2.2 (number: 0–3) and non-implanted was 2.6 (number: 2–4) per mouse (Fig. 2a and b).

Compared with pre-xenografted myomas and non-implanted myomas, the implanted myomas possessed more expressions of cell density, ERα, PR, vimentin, VEGF, MVD, and Ki-67 labeling index (Fig. 2c–j). In addition, the expression of SMA was similar in the groups of pre-xenografted and implanted myomas but decreased in non-implanted myomas. Thus, similar SMA expressions in the groups of pre-xenografted and implanted myomas confirmed the establishment of PM growth in this laparoscopically-induced PM mouse model which were presented from the patient with PMs and in situ UM as well (Additional file 5: Figure S3; Additional file 1: Table S3).

In conclusion, implanted myomas had more cellularity, sex steroid receptor expressions, angiogenesis and proliferative property compared with pre-xenografted or non-implanted myomas.

### Depletion of oestrogen decreased laparoscopically-induced PM implantations

**Fig. 3:**
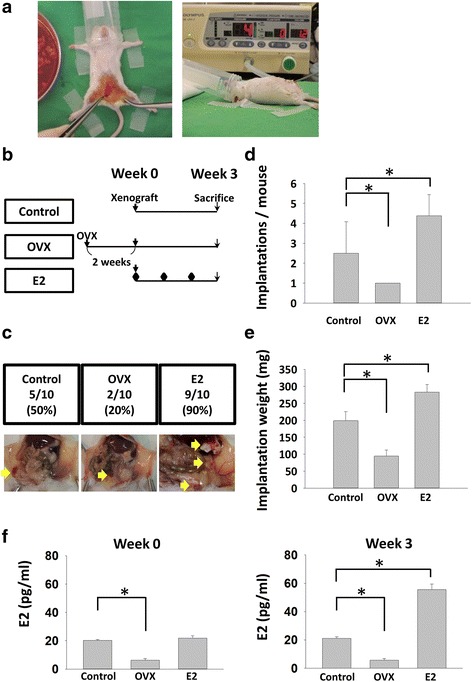
Oestradiol (E2) increased implantation numbers and implantation weight per mouse and depletion of oestrogen had contrary results. **a** Xenograft and insufflation procedures. **b** and **c** Flow chart and representative pictures of the xenografted PM mouse model. Mice were sacrificed in 3 weeks after xenograft procedures. **d** and **e** Compared with control group, there were fewer implantations and weight of implantations in the OVX group, while there were more implantations and weight of implantations in the E2 group. **f** E2 levels of control, OVX and E2 groups at Week 0 and 3. Implantations/mouse stands for implanted uterine myoma numbers per mouse at sacrifice; implantation weight (mg) stands for the total weight of implanted uterine myoma per mouse (* *P* < 0.05)

Then we investigated whether the implantations of PM lesions correlated with serum E2 levels. At week 3, sacrificed OVX group mice had significantly less implantations and weight per mouse compared with control group. By comparison, mice in E2 group had more total implantation numbers and weight compared with control group (Fig. 3b–e). At Week 0, E2 levels of OVX group were significantly lower compared with control group, while no statistical difference between control and E2 groups. At Week 3, significantly higher E2 levels of E2 group were demonstrated (Fig. 3f).

**Fig. 4:**
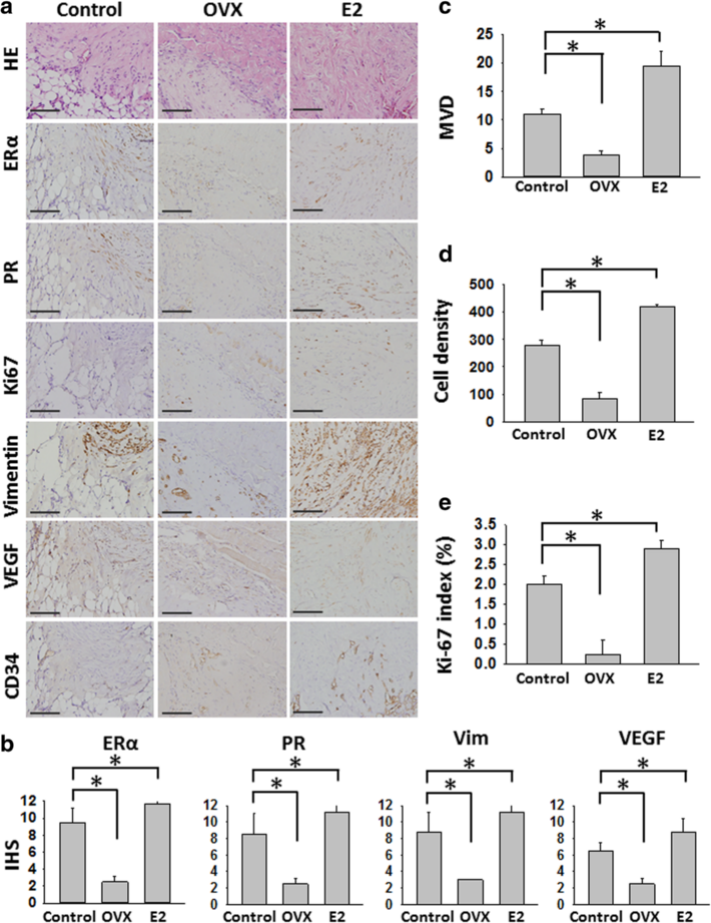
E2 was associated with increasing cell density, ERα, PR, cytoskeletal proteins and angiogenesis expressions in the xenografted mice model. **a** Staining for H&E, and IHC for ERα, PR, Ki67, vimentin, VEGF, and CD34 of implanted myoma in the three groups (Control, OVX and E2 groups). Original magnification: ×400; the scale bars represent 400 μm; **b** IHS of the samples of implanted myoma. **c**–**e** Compared with Control group, there were less MVD, cell density and Ki-67 index in the OVX group (*P < 0.05), while there were more MVD, cell density and Ki-67 index in the E2 group (* *P* < 0.05). OXV = ovariectomy. **P < 0.01

The harvested implanted myomas of OVX group possessed lower cellularity, ERα, PR, Ki-67, vimentin and MVD expressions than those of control group. By contrary, cellularity, ERα, PR, Ki-67, vimentin and MVD expressions significantly increased in the E2 group (Fig. 4a–e & IHS listed in Additional file 1: Table S4).

This result demonstrated that additional E2 increased the implantations, sex steroid receptor expressions, proliferation and angiogenesis while depletion of oestrogen had contrary results.

### Hormonal manipulation in laparoscopically-induced PM mouse model

**Fig. 5:**
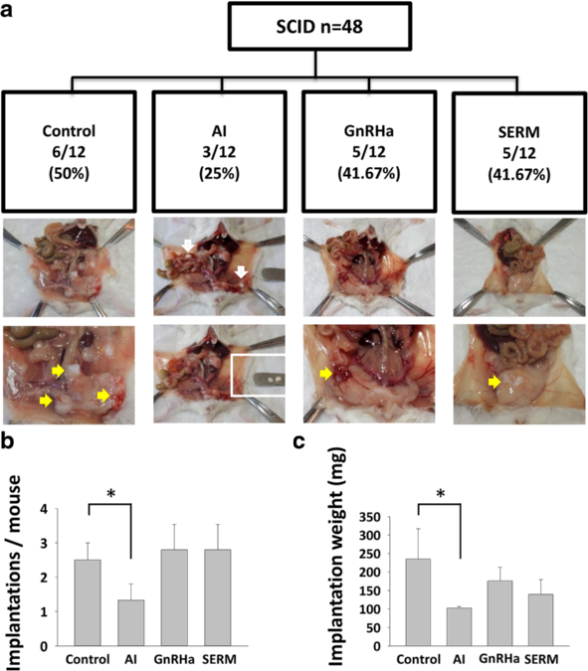
Therapeutic effects of sex hormone modulators in the laparoscopically-induced PM mouse model. **a** Flow chart and representative pictures of the laparoscopically-induced PM mouse model and sex hormone manipulations- Control, AI, GnRHa, and SERM. *Yellow arrows* indicated implanted myoma. *White box* indicates non-implanted myoma. **b** and **c** Average implantations per mouse and implantation weight per mouse in each group. Compared with control group, AI significantly decreased the implantations and implantation weight per mouse. *P < 0.05

**Fig. 6:**
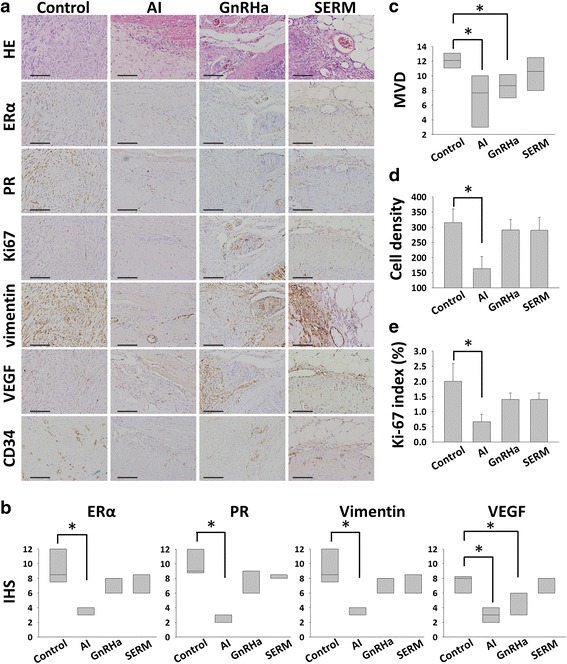
Results of H&E and IHC of the 4 groups (Control, AI, GnRHa, and SERM groups). **a** H&E and IHC stainings for ERα, PR, Ki67, vimentin, VEGF, and CD34 of the samples of implanted myoma. Original magnification: ×400; the scale bars represent 400 μm. **b** AI significantly decreased expressions of ERα, PR, and vimentin. **c** AI and GnRHa inhibited the angiogenesis (VEGF expression and MVD). **d** and **e** Cell density and Ki-67 index were decreased in AI group. *P < 0.05, **P < 0.01

Finally, we tested and analysed if the hormonal manipulation could inhibit establishment of PM lesions. After 3-week treatment with three different ligands, AI significantly decreased the implantation numbers and implantation weight compared with control group (Fig. 5a and b). The expressions of cellularity, ERα, PR, vimentin, VEGF, MVD, and Ki67 index were also significantly decreased in AI treatment group (Fig. 6a–e & Additional file 5: Figure S3). In addition, GnRHa significantly inhibited the angiogenesis expressions (VEGF and MVD). However, both GnRHa and SERM lowered the implantations, weight of implantation, and Ki-67 labeling index while these results were not significant (Fig. 6a–e & Additional file 1: Table S5).

## Discussion

Until now, the treatment and prevention of laparoscopically-induced PMs are still challenges for clinical physicians. In this study, we employed a new animal model to examine the role of oestrogen in laparoscopically-induced PMs. First, implanted myomas possessed more ERα, PR, angiogenesis and proliferative property compared with pre-xenografted or non-implanted myomas. Second, depletion of oestrogen significantly decreased laparoscopically-induced PM implantations. The implantations, angiogenesis, and proliferative property of PMs were associated with the serum E2 levels. Third, sex steroid hormone modulator, AI, decreased the implantations, angiogenesis, and proliferative property. To our knowledge, this is the first report to demonstrate the involvement of oestrogen-induced angiogenesis in the development of PMs. These data highlighted the crucial role of oestrogen-induced angiogenesis and implantations in the development of PMs, and hormonal manipulation with AI might be potential remedies in preventing laparoscopically-induced PMs.

Research of UMs is still challenged by the need for in vivo models. Until now, the laparoscopically-induced PMs in vivo model is lacking. At present, the commonly used transplantation model for myomas is the ELT3 cell injection model. Owing to a germ-line mutation of the tuberous sclerosis gene 2 (Tsc2), Eker rats spontaneously develop various neoplasms, including leiomyosarcoma [38–40]. Nonetheless, the Tsc2-driven tumour development might not reflect the sporadic pathogenesis of myomas. An alternative model described by Hassan et al. [41], who provoked growth of myoma-like xenograft implanted subcutaneously in SCID mice, by transplanting human tumour tissue pieces overexpressing COX2 (cyclooxygenase 2) and VEGF through adenoviral transduction. However, that model exhibits substantial limitations because it is driven by the ectopic overexpression of COX2 [42–44]. In recently, Drosch et al. report an approach by generating a myoma xenograft model through injection of human myoma–derived primary cells without genetic manipulation into myometrium of mice [45]. According to Drosch’s report, primary myoma cells are suited to generate fibroid-like xenografts for studying pathogenesis without genetic modifications [45]. Furthermore, one single stem cell is thought to give birth to a specific myoma (which is why it is called a clonal disease) [46]. Thus, it is reasonable that human UM xenografts are suited to generate PMs-like xenografts model without genetic modifications. For the first time, we could demonstrate this laparoscopically-induced PMs and setup a pneumoperitoneum system in SCID mice. SCID mice possess a combined congenital deficiency in T- and B-lymphocyte function, and cannot reject transplanted non-autologous tissues [32, 47]. Those tumours closely mimicked laparoscopically-induced PMs by their highly similar histology and growth characteristics. The xenografted tissues can be used to simulate aspects of the myoma-host microenvironment during the pathogenesis of PMs. Moreover, using different donors would also enhance the interpatient variability. Aside from these restrictions, the described model provides a powerful tool for basic research to investigate the pathogenesis of PMs, because this model omits any genetic modifications and closely resembles the pathogenesis of laparoscopically-induced PMs. This model can be used as a powerful tool to study mechanisms involved in the pathogenesis of laparoscopically-induced PMs and will open new opportunities for their treatment.

Although quite a lot is known about the factors contributing to myoma growth, the pathophysiology of PMs remains largely unelucidated [1]. Traditionally, PMs were supposed to be pedunculated subserosal myomas that were accidentally separated from the uterus, and subsequently attached to other organs in the pelvis for the provision of their blood supply [48]. Since the published case reports of PM after laparoscopic myomectomy, it was hypothesised that iatrogenic PMs could develop by seeding of retained small tissue fragments with stem cell after morcellation in the peritoneal cavity [22, 49]. Our result showed that implanted xenografts similar to primary UM histologically. Xenografts showed features of UM-like fusiform cells: a whirl-like pattern of the smooth muscle bundles and cigar-shaped nuclei determined by histology (Fig. 2c). In addition, SMA showed a strong abundance in the implanted xenografts and in the primary tumours (Additional file 5: Figure S3), confirming the smooth muscle differentiation of the xenografts. According to our case report and animal model, angiogenesis and cell proliferation increased in implanted xenografted myoma compared with primary or non-implanted xenografted myoma. Of note, Ishikawa et al. showed that the growth of tissue xenografts was strongly dependent on the cell density of the xenografts [22]. Hassan et al. showed VEGF provoked growth of myoma-like xenografts implanted subcutaneously in SCID mice [41]. VEGF-enhanced angiogenesis is also associated with an increase in vascular permeability, which results in an increase in the amount of growth factors and nutrients delivered to tumour cells [44, 50]. Thus, implantation, angiogenesis and cell proliferation are the important mechanisms for laparoscopically-induced PM growth.

UM are oestrogen- and progesterone-dependent monoclonal tumours that arise from the uterine smooth muscle tissue [51]. Data from in vitro and nonhuman animal models over decades suggest that E2 plays a central role in myoma growth via its receptor, ERα [28, 46]. Furthermore, the impact of sex hormones on the PM growth was illustrated by cases of rapid growth of PMs during pregnancy. Cucinella et al. [8] report a case of a woman where an asymptomatic PM was discovered during a cesarean section, 24 months after the procedure of laparoscopic myomectomy. Takeda et al. [52] published a case report of a woman who was diagnosed with a PM 2 years after the procedure of laparoscopic myomectomy. After 2 years of conservative treatment, the size of the mass remained the same, yet during pregnancy, the rapid growth of this mass was observed, supporting the sex hormonal impact on the growth of PM. In our study, the non-implanted myoma fragments disclosed decreased expressions of both ERα and PR compared with implanted lesions, and this result suggested the disseminated fragments with strong positive ERα and PR might be essential in the development of PM implantations. Our study also demonstrated that depletion of oestrogen decreased the angiogenesis, proliferation and implantations, and the result was correlated with previous studies which demonstrated shrinkage of benign metastatic leiomyomatosis after bilateral salpingo-oophorectomy (BSO) surgery [53–56]. The duration of sex steroid hormone exposure after laparoscopic morcellation might also be a risk factor for the development of PMs [26]. According to the systematic review, 69 cases PMs after laparoscopic myomectomy by Meulen et al., it is hypothesised that prolonged exposure to sex steroid hormones such as hormonal replacement therapy, could be a risk factor for the development of PMs [8, 16, 23, 31, 57]. Our present study highlighted the crucial role of E2 and ERα and PR expressions in the development of laparoscopically-induced PMs.

To prevent PMs, it is important not to disperse tissue fragments during intra-corporeal power morcellation and to inspect all the surgical fields during laparoscopy with caution [58, 59]. Until now, no effective method to prevent tissue dispersion from power morcellation has been established [60–62]. Most patients with symptomatic PMs after laparoscopic surgeries at their reproductive age, and BSO might not be indicated in all patients for the purpose of prevention of PMs [63, 64]. Thus it is necessary to find medical prevention and treatment for those patients. Leiomyomatosis peritonealis disseminata (LPD) is a disease that is characterised by the presence of many (sub-) peritoneal smooth-muscle nodules disseminated through the omentum and peritoneum. The nodules are thought to originate from metaplasia of subperitoneal mesenchymal stem cells without a history of laparoscopic myomectomy. Although LPD is a different entity when compared with UMs, there might be a relation between morcellation of UMs and an iatrogenic development of this disease. Several studies which presented that LPD shrink under sex steroid hormone manipulation, including BSO [53–55], menopause [65], megestrol [66], SERM [55], AI [55, 67], and GnRHa [55, 56, 68]. Laparoscopic myomectomy might result in the growth of multiple PM nodules mimicking LPD [31]. Thus it is reasonable to test the prevention and therapeutic effect of sex hormone manipulation on PMs after laparoscopic surgery.

Our xenografted mouse model demonstrated that AI significantly decreased the implantations rate and weight of implantations, while GnRHa and SERM did not change the implantation rate.

Aromatase is a cytochrome P450 enzyme (CYP-19) that allows the transformation of androgens into oestrogens. AIs are compounds that interact with the hormone-binding site of the molecule (exemestane) or with its catalytic subunit (anastrozole, letrozole). This is supported mostly by clinical data showing that AIs are as effective as GnRH analogues in reducing myoma volume in premenopausal women. Letrozole reduces fibroid volumes by 46 % (vs. 32 % in the GnRH analogue group) [69]. In peripheral tissues, including skin and adipose tissue, and the ovaries, aromatase catalyses the formation of oestrogen, which reaches UM tissue through the circulation. In addition, aromatase in myoma tissue converts androstenedione of adrenal or ovarian origin to oestrogen locally [70]. This is possible reason AI is more effect than GnRHa in our study.

After binding to the GnRH receptors, GnRHa induces subsequent stimulation of gonadotropin secretion followed by desensitisation, and thus they delay the gonadotropic axis blockade. In vitro, GnRHa inhibits cell proliferation and induces apoptosis [71]. Even though, GnRHa is the most effective agent to correct anaemia, and reduce fibroid volume by 50 % after 2–3 months treatment [1, 2]. However, in our study, GnRHa did not reach the comparable results with AI and SPRM in lowering uterine myoma implantations. The possible reason was that because GnRHa takes approximately 1–3 weeks to obtain a fully hypogonadotropic hypogonadal state to decrease serum E2 level [72, 73], while we sacrificed mice only after 3 weeks GnRHa treatment. Thus, further study of the suitable timing of GnRHa treatment for PMs is needed.

SERMs are nonsteroidal oestrogen receptor ligands with agonist or antagonist effects depending on the tissue. Raloxifene (SERM) has been approved for the treatment and prevention of post-menopausal osteoporosis [74]. Three randomised controlled trials have evaluated raloxifene in premenopausal women with confirmed UMs [75]. Two of these trials, including 215 women, showed the therapeutic efficacy of raloxifene, but the third did not. This may be due to the rise in E2 secretion observed in premenopausal women following SERM treatment. There was no therapeutic effect on PMs in our study.

Our study had some limitations, including lack of long-term follow-up of treatment of different ligands, and lack of serum hormone level changes of progesterone. In addition, we did not examine the expression of the somatic mutations in the mediator complex subunit 12 (MED12) gene which might be associated with 50 % of UM [76].

## Conclusions

Until now, there is still no effective method to treat and prevent PMs after laparoscopic power morcellation. The identification of tumourigenic factors will give insights into the pathogenesis of UMs and might open new possibilities for drug testing. Based on the present study, PMs are oestrogen-dependent and AI might be potential remedies to prevent PMs after laparoscopic procedures.

## Additional files

Additional file 1: Table S1. Patient characteristics. **Table S2**. List of proteins tested by antibodies and characteristics of the corresponding antibodies used. **Table S3**. IHS of pre-xenografted myoma and implanted parasitic myoma. **Table S4**. IHC scores of control, OVX and E2 groups. **Table S5**. IHC scores of control, AI, GnRHa, and SERM. (DOCX 33 kb)
Additional file 2: Figure S1. Surgical procedures. (A) Pneumoperitoneum was simulated with Surgineedle™ for 10 min after xenograft procedure and pressure setting was 4 mmHg. (B) Procedure of ovariectomy (OVX) of SCID mice. (TIF 470 kb)
Additional file 3: Supplemental Material and Methods. **Part 1**. Laparoscopically-induced parasitic myoma model: Xenograft of human uterine myoma in SCID mice. **Part 2**. Serum E2 levels of SCID mice. **Part 3**. Histological and immunohistochemistry (IHC) analysis. (DOC 52 kb)
Additional file 4: Figure S2. Cell density and IHS of ERα, PR, SMA, Ki67, vimentin, VEGF, and CD34 in samples of in situ uterine myoma (UM) and parasitic myoma (PM) in patient No. 1. The bars show the mean value ± standard deviation. (TIF 83 kb)
Additional file 5: Figure S3. SMA expression in pre-xenografted, implanted and non-implanted myomas; the scale bars represent 400 μm. (TIF 210 kb)

## Abbreviations

AI: Aromatase inhibitor
BSO: Bilateral salpingo-oophorectomy
COX2: Cyclooxygenase 2
DMEM: Dulbecco’s modified eagle’s medium
E2: 17β-oestradiol
ELT3 cell: Eker leiomyoma tumor-3 cell
ER: Estrogen receptor
FBS: Foetal bovine serum
GnRHa: Gonadotrophin-releasing hormone agonists
IHC: Immunohistochemistry
IHS: Immunohistochemical scores
LM: Laparoscopic myomectomy
MVD: Microvessel density
NOD-SCID: Non-obese diabetic severe combined immunodeficiency
OVX: Ovariectomised
PM: Parasitic myoma
PR: Progesterone receptor
UM: Uterine myoma
VEGF: Vascular endothelial growth factor
