# Supplementary material for: Oestrogen-induced angiogenesis and implantation contribute to the development of parasitic myomas after laparoscopic morcellation
Source: Reprod Biol Endocrinol. 2016 Oct 6;14:64. doi: 10.1186/s12958-016-0200-y (PMC5053344; doi:10.1186/s12958-016-0200-y)
Supplement: Additional file 1: Table S1. — Patient characteristics. Table S2. List of proteins tested by antibodies and characteristics of the corresponding antibodies used. Table S3. IHS of pre-xenografted myoma and implanted parasitic myoma. Table S4. IHC scores of control, OVX and E2 groups. Table S5. IHC scores of control, AI, GnRHa, and SERM. (DOCX 33 kb) [file 12958_2016_200_MOESM1_ESM.docx]

**Table S1**

**Patient characteristics**

| **Case No.** | **1*** | **2** | **3** | **4** | **5** | **6** | **7** | Mean ± SD |
| --- | --- | --- | --- | --- | --- | --- | --- | --- |
| **Surgery**  **(H/M)** | H | H | H | M | M | H | H |  |
| **Age** | 44 | 42 | 45 | 41 | 39 | 43 | 46 | 42.86±2.23 |
| **Parity** | 2 | 1 | 3 | 0 | 1 | 2 | 3 | 1.8±1.17 |
| **Myoma No.** | 6 | 6 | 12 | 5 | 7 | 8 | 10 | 8.4±2.42 |
| **Largest (cm)** | 5 | 6.5 | 4.5 | 6 | 7 | 6.5 | 4.5 | 5.7±1.03 |
| **Hgb (gm/dl)**** | 10.0 | 10.3 | 9.6 | 9.4 | 8.1 | 9.3 | 8.8 | 9.36±0.68 |
| **CA125 (U/ml)**** | 33.65 | 25.78 | 21.10 | 12.69 | 9.83 | 36.82 | 42.65 | 20.61±8.67 |
| **Pain score***** | 10 | 6 | 4 | 5 | 8 | 8 | 7 | 6.6±2.15 |

Abbreviation: H: hysterectomy; M: myomectomy. *Patient with parasitic myomas. **Preoperative study; *** Pain score, as a visual analog pain scale (VAS), consisted of a non-graduated 10-cm line ranging from “no pain” to “pain as bad as it could be.

**Table S2**

**List of proteins tested by antibodies and characteristics of the corresponding antibodies used**

| Protein | Assay | Antibody | Origin | Dilution | Incubation period |
| --- | --- | --- | --- | --- | --- |
| **ERα** | IHC | Clone 1D5, M7074 Dako | mmab | 1/100 | 30 mins, RT |
| **PR** | IHC | Clone PgR636, Dako | mmab | 1/200 | 30 mins, RT |
| **SMA** | IHC | M0581, DAKO | mmab | 1/500 | 30 mins, RT |
| **Ki-67** | IHC | RM-9106-RQ, Dako | rmab | 1/100 | 30 mins, RT |
| **Vimentin** | IHC | Clone V9, M0725, DAKO | mmab | 1/2000 | 30 mins, RT |
| **VEGF** | IHC | A20SC-152, Santa Cruz | rpab | 1/200 | 2 hours, RT |
| **CD34** | IHC | Clone QBEnd10, M7165, Dako | mmab | 1/500 | 30 mins, RT |

Abbreviations: IHC, immunohistochemistry; mmab, mouse monoclonal antibody; rpab, rabbit polyclonal antibody; RT, room temperature.

**Table S3**

**IHS of pre-xenografted myoma and implanted parasitic myoma**

|  | a | b | c | a v.s. b | b v.s. c |
| --- | --- | --- | --- | --- | --- |
|  | Pre-xenografted myoma (UM) | Implanted myoma | Non-implanted myoma |  |  |
| **Cell density** | 188.67 ± 32.33 | 315.20 ± 44.90 | 92.80 ± 21.16 | 0.04 | 0.03 |
| **ER α** | 7.60 ± 1.52 | 9.40 ± 1.52 | 1.14 ± 0.51 | 0.097 | <0.01 |
| **PR** | 6.20 ± 2.28 | 9.20 ± 1.63 | 3.40 ± 0.89 | 0.044 | <0.01 |
| **SMA** | 7.80 ± 2.16 | 8.00 ± 2.45 | 4.20 ± 1.01 | 0.859 | <0.01 |
| **Ki-67 (%)** | 1.50 ± 0.50 | 2.30 ± 0.57 | 0.30 ± 0.27 | 0.045 | <0.01 |
| **Vimentin** | 8.00 ± 1.23 | 10.20 ± 1.64 | 2.60 ± 1.14 | 0.043 | <0.01 |
| **VEGF** | 4.00 ± 1.41 | 7.00 ± 2.00 | 2.00 ± 1.00 | 0.035 | <0.01 |
| **CD34 (MVD)** | 7.33 ± 1.56 | 10.40 ± 1.67 | 3.00 ± 0.62 | 0.017 | <0.01 |

**Table S4**

**IHC scores of control, OVX and E2 groups**

|  | **a** | **b** | **c** | **a v.s. b** | **a v.s. c** |
| --- | --- | --- | --- | --- | --- |
|  | **Control** | **OVX** | **E2** |  |  |
| **Implantation** | 2.50 ± 1.58 | 1.00 ± 0 | 4.38 ± 1.06 | <0.01 | <0.01 |
| **Weight (mg)** | 199.1 ± 26.67 | 94.6 ± 8.12 | 283.2 ± 22.31 | <0.01 | 0.011 |
| **Cell density** | 280 ± 18.26 | 83.5 ± 21.2 | 420 ± 8.77 | 0.036 | 0.028 |
| **ER α** | 9.50 ± 1.73 | 2.50 ± 0.71 | 11.63 ± 1.06 | *<* 0.01 | 0.023 |
| **PR** | 8.50 ± 2.52 | 2.50 ± 0.71 | 11.25 ± 1.39 | 0.035 | 0.032 |
| **Ki-67 (%)** | 2.0 ± 0.20 | 0.25 ± 0.35 | 2.88 ± 0.23 | < 0.01 | 0.01 |
| **Vimentin** | 8.75 ± 2.50 | 3.0 ± 0 | 11.25 ± 1.39 | 0.037 | 0.046 |
| **VEGF** | 6.50 ± 1.0 | 2.50 ± 0.71 | 8.75 ± 1.67 | <0.01 | 0.034 |
| **CD34 (MVD)** | 11.04 ± 0.85 | 3.83 ± 0.71 | 19.50 ± 2.56 | < 0.01 | < 0.001 |

**Table S5**

**IHC scores of control, AI, GnRHa, and SERM**

|  | a | b | c |  | e |  | a v.s b |  |
| --- | --- | --- | --- | --- | --- | --- | --- | --- |
|  | Control | AI | GnRHa |  | SERM |  |  |  |
| **Implantation** | 2.50±0.50 | 1.33±0.47 | 2.80±0.75 |  | 2.80±0.75 |  | 0.021 |  |
| **Weight** | 234.98±82.18 | 102.73±3.58 | 175.90±37.62 |  | 139.48±39.60 |  | 0.044 |  |
| **Cell density** | 315.20±44.90 | 163.33±40.72 | 204.67±28.50 |  | 241.55±66.75 |  | <0.001 |  |
| **ER α** | 9.33±2.13 | 3.0±0.81 | 8.0±3.35 |  | 5.20±0.98 |  | < 0.01 |  |
| **PR** | 9.83±1.57 | 2.33±0.47 | 7.2±2.4 |  | 6.4±1.50 |  | < 0.01 |  |
| **Ki-67 (%)** | 2±0.58 | 0.67±0.24 | 0.9±0.2 |  | 1.2±0.4 |  | 0.012 |  |
| **Vimentin** | 9.16±2.19 | 3.33±0.47 | 6.00±1.26 |  | 7.00±1.26 |  | 0.012 |  |
| **VEGF** | 7.50±1.19 | 3.00±0.82 | 4.80±1.64 |  | 6.80±0.98 |  | <0.01 |  |
| **CD34 (MVD)** | 12.02±1.05 | 6.89±2.91 | 8.60±1.64 |  | 9.78±1.14 |  | 0.012 |  |

Implantation: implanted myomas per mouse; Weight: implantation weight per mouse.; VEGF a v.s. c, *P*=0.012; MVD a v.s. c, *P*=0.004.
