# Supplementary figures and images for: Oestrogen-induced angiogenesis and implantation contribute to the development of parasitic myomas after laparoscopic morcellation
Source: Reprod Biol Endocrinol. 2016 Oct 6;14:64. doi: 10.1186/s12958-016-0200-y (PMC5053344; doi:10.1186/s12958-016-0200-y)

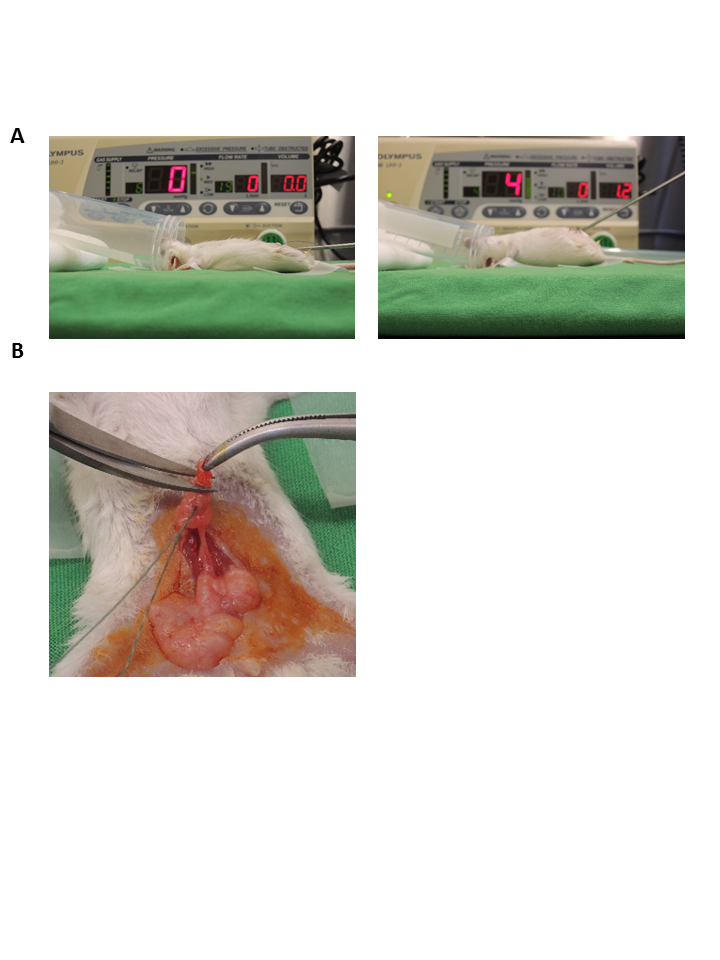

Supplement: Additional file 2: Figure S1. — Surgical procedures. (A) Pneumoperitoneum was simulated with Surgineedle™ for 10 min after xenograft procedure and pressure setting was 4 mmHg. (B) Procedure of ovariectomy (OVX) of SCID mice. (TIF 470 kb) [file 12958_2016_200_MOESM2_ESM.tif]

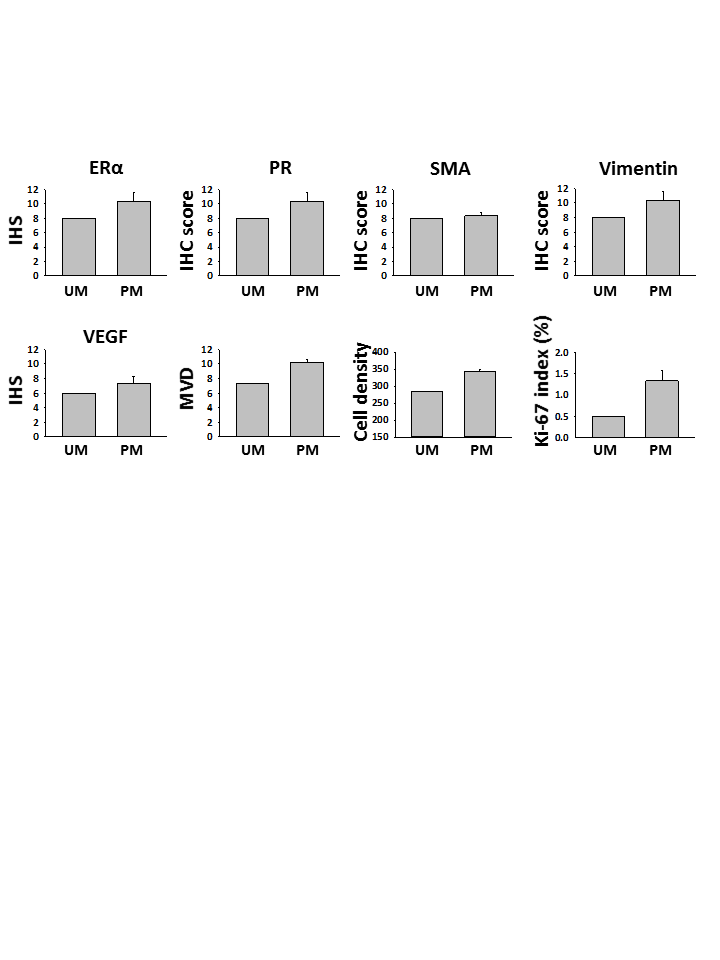

Supplement: Additional file 4: Figure S2. — Cell density and IHS of ERα, PR, SMA, Ki67, vimentin, VEGF, and CD34 in samples of in situ uterine myoma (UM) and parasitic myoma (PM) in patient No. 1. The bars show the mean value ± standard deviation. (TIF 83 kb) [file 12958_2016_200_MOESM4_ESM.tif]

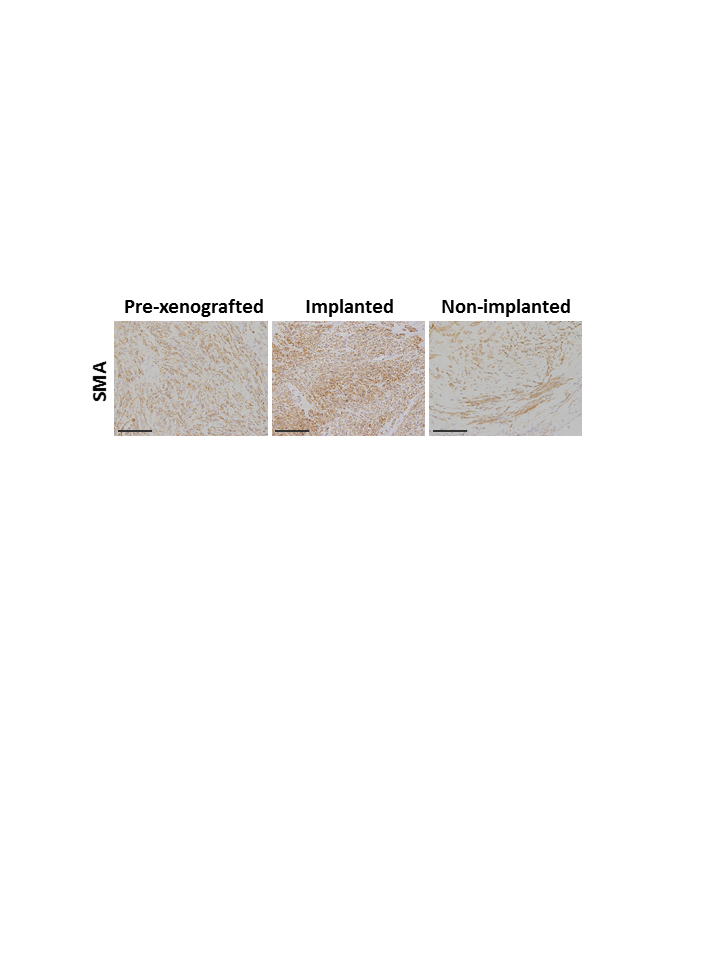

Supplement: Additional file 5: Figure S3. — SMA expression in pre-xenografted, implanted and non-implanted myomas; the scale bars represent 400 μm. (TIF 210 kb) [file 12958_2016_200_MOESM5_ESM.tif]
