# Supplementary material for: Oestrogen-induced angiogenesis and implantation contribute to the development of parasitic myomas after laparoscopic morcellation
Source: Reprod Biol Endocrinol. 2016 Oct 6;14:64. doi: 10.1186/s12958-016-0200-y (PMC5053344; doi:10.1186/s12958-016-0200-y)
Supplement: Additional file 3: — Supplemental Material and Methods. Part 1. Laparoscopically-induced parasitic myoma model: Xenograft of human uterine myoma in SCID mice. Part 2. Serum E2 levels of SCID mice. Part 3. Histological and immunohistochemistry (IHC) analysis. (DOC 52 kb) [file 12958_2016_200_MOESM3_ESM.doc]

**Supplemental Material and Methods**

List and numbering of each element (part 1-3)

**Part 1. Laparoscopically-induced parasitic myoma model: Xenograft of human uterine myoma in SCID mice.** (Page 2)

**Part 2. Serum E2 levels of SCID mice.** (Page 3)

**Part 3. Histological and immunohistochemistry (IHC) analysis.** (Page 4-5)

**Part 1**

**Laparoscopically-induced parasitic myoma model: Xenograft of human uterine myoma in SCID mice**

General anesthesia of these SCID mice was performed with 4% isoflurane and maintained them on 1.5% isoflurane anesthesia ^1^. After the xenograft procedure, abdominal wall in two layers using a continuous 3-0 chromic catgut suture for peritoneum and an interrupted 3-0 vicry l suture for the skin. The pneumoperitoneum was induced by using Olympus UHI-3 high flow insufflator (Olympus, Japan). The CO2 insufflation pressure was 4 mmHg and the duration of insufflation was 10 minute ^2^. These mice were left untreated and sacrificed in 3 weeks after the xenotransplantation. Both implanted and non-implanted fragments were harvested.

**Part 2**

**Serum E2 levels of SCID mice**

The serum collection, 50μl from tail vein of each mouse, were performed at week 0 (just before xenograft procedure) and week 3 (just before sacrifice) into tubes precoated with potassium-EDTA, and centrifuged at 3000g and 4 °C for plasma preparation. Serum E2 levels were assessed using the mouse E2 ELISA kit (ES180S-100, Calbiotech, U.S.) according to the manufacturer’s instructions ^3^.

**Part 3**

**Histological and immunohistochemistry (IHC) analysis**

The H&E and IHC staining were performed. The slides incubated with isotype-matched immunoglobulin G from normal rabbits were applied as a negative control. For the positive control, we used the breast cancer samples that were positive for ERα and PR. All immunoreactions were detected with diaminobenzidine (DAB). The histological and immunohistochemistry slides were independently examined by two observers. The cell density was determined on H&E sections by counting the number of nuclei per optical field with a X20 objective lens ^4^. We utilized immunohistochemical scores (IHS) based on the German ImmunoReactive score ^5^. The IHS was calculated by combining an estimate of the percentage of immunoreactive cells (quantitative score) with an estimate of the staining intensity (staining intensity score). No staining was scored as 0; staining of 1–10% as 1; 11–50% as 2; 51–80% as 3; and 81–100% as 4. The staining intensity was rated on a scale of 0 to 3, with 0 representing no staining, 1 weak staining, 2 moderate staining, and 3 strong staining. The raw data were converted to IHS by multiplying the quantity score and the intensity score. A moderate to strong level (IHS > 4) of ERα, PR, SMA, vimentin, and VEGF expression was considered to be positive, and weak or absent expression (IHS 0–4) was considered to be negative.

The Ki-67 labeling index was defined as the percentage of positively labeled cells ^6^. Microvessel density (MVD) was determined as follows. The highly vascularized areas of the tumour stained with a CD34 antibody were identified and CD34-positive microvessels were counted within a high-power field (number per 0.75 mm^2^) ^7^. Single endothelial cells or clusters of endothelial cells, with or without lumen, were considered individual vessels. MVD was expressed as the vessel number/high-power field in sections. Three fields were counted per animal and the average was taken as the MVD of each tumor.

**References**

1. Raoul C, Abbas-Terki T, Bensadoun JC, Guillot S, Haase G, Szulc J, et al. Lentiviral-mediated silencing of SOD1 through RNA interference retards disease onset and progression in a mouse model of ALS. Nat Med. 2005 Apr;11(4):423-8.

2. Carter JJ, Feingold DL, Kirman I, Oh A, Wildbrett P, Asi Z, et al. Laparoscopic-assisted cecectomy is associated with decreased formation of postoperative pulmonary metastases compared with open cecectomy in a murine model. Surgery. 2003 Sep;134(3):432-6.

3. Haisenleder DJ, Schoenfelder AH, Marcinko ES, Geddis LM, Marshall JC. Estimation of estradiol in mouse serum samples: evaluation of commercial estradiol immunoassays. Endocrinology. 2011 Nov;152(11):4443-7.

4. Ishikawa H, Ishi K, Serna VA, Kakazu R, Bulun SE, Kurita T. Progesterone is essential for maintenance and growth of uterine leiomyoma. Endocrinology. 2010 Jun;151(6):2433-42.

5. Remmele W, Schicketanz KH. Immunohistochemical determination of estrogen and progesterone receptor content in human breast cancer. Computer-assisted image analysis (QIC score) vs. subjective grading (IRS). Pathol Res Pract. 1993 Sep;189(8):862-6.

6. Lopez F, Belloc F, Lacombe F, Dumain P, Reiffers J, Bernard P, et al. Modalities of synthesis of Ki67 antigen during the stimulation of lymphocytes. Cytometry. 1991;12(1):42-9.

7. Huang TS, Chen YJ, Chou TY, Chen CY, Li HY, Huang BS, et al. Oestrogen-induced angiogenesis promotes adenomyosis by activating the Slug-VEGF axis in endometrial epithelial cells. Journal of cellular and molecular medicine. 2014 Jul;18(7):1358-71.
